# Role of Interleukin-17 in Acute Pancreatitis

**DOI:** 10.3389/fimmu.2021.674803

**Published:** 2021-09-14

**Authors:** Guanqun Li, Hongze Chen, Liwei Liu, Peng Xiao, Yu Xie, Xinglong Geng, Tao Zhang, Yang Zhang, Tianqi Lu, Hongtao Tan, Le Li, Bei Sun

**Affiliations:** ^1^Department of Pancreatic and Biliary Surgery, The First Affiliated Hospital of Harbin Medical University, Harbin, China; ^2^Key Laboratory of Hepatosplenic Surgery, Ministry of Education, Harbin, China

**Keywords:** interleukin-17, acute pancreatitis, T helper 17 cells, immune response, gut microbiome

## Abstract

Acute pancreatitis (AP) is a leading cause of death and is commonly accompanied by systemic manifestations that are generally associated with a poor prognosis. Many cytokines contribute to pancreatic tissue damage and cause systemic injury. Interleukin-17 (IL-17) is a cytokine that may play a vital role in AP. Specifically, IL-17 has important effects on the immune response and causes interactions between different inflammatory mediators in the AP-related microenvironment. In this literature review, we will discuss the existing academic understanding of IL-17 and the impacts of IL-17 in different cells (especially in acinar cells and immune system cells) in AP pathogenesis. The clinical significance and potential mechanisms of IL-17 on AP deterioration are emphasized. The evidence suggests that inhibiting the IL-17 cytokine family could alleviate the pathogenic process of AP, and we highlight therapeutic strategies that directly or indirectly target IL-17 cytokines in acute pancreatitis.

## Background

Acute pancreatitis (AP) is an inflammatory disorder of the pancreas related to tissue damage and includes a cascade of adverse cellular events (1, 2). Starting from the initial premature activation of enzymes with the involvement of the immune system in a potential systemic inflammatory reaction and organ failure, acute pancreatitis has a high lethality and poor prognosis. Currently, there is no effective therapeutic agent that reduces the risks and consequences of AP, which has a mortality rate of up to 30% (3, 4). Over the past decade, immune system activation has been identified as a key trigger and regulator of inflammatory injury in the pancreas, affecting the extent of pancreatic necrosis, organ failure and disease deterioration (5, 6). AP leads to overactivation of leukocytes and increased neutrophil metastasis to inflammation, with a consequent release of proinflammatory factors, including several forms of leukocyte interleukins, procalcitonin, transforming growth factor, and tumor necrosis factor (7, 8). The elevated expression of cytokines seems to provide objective evidence in the progression of AP and is paralleled by a pronounced immune response that amplifies disease severity.

Interleukin-17 (IL-17) is an important proinflammatory cytokine produced by T helper 17 (Th17) cells, γδ T cells and natural killer (NK) cells (9). The primary function of IL-17 is to mediate responses to pathogens and symbioses through various targets, all of which balance the inflammatory response with the immune system (10). In addition to its potential role in regulating the immune response to balance cytotoxic and tolerant immune profiles, it also results in acute injury. As a proinflammatory cytokine, IL-17 essentially incorporates a complicated network of cytokines that is responsible for various inflammatory conditions and pathogenesis (11–14). The inflammatory response and cytokine production are particularly crucial in the progression of AP. To date, few studies have systematically described the role of the IL-17 family in AP. This review outlines the biological properties of IL-17 and its impacts on the pathogenesis of AP. Our review proposes to broaden the treatment of AP by targeting IL-17.

## IL-17 Family and Receptors

**Figure 1:**
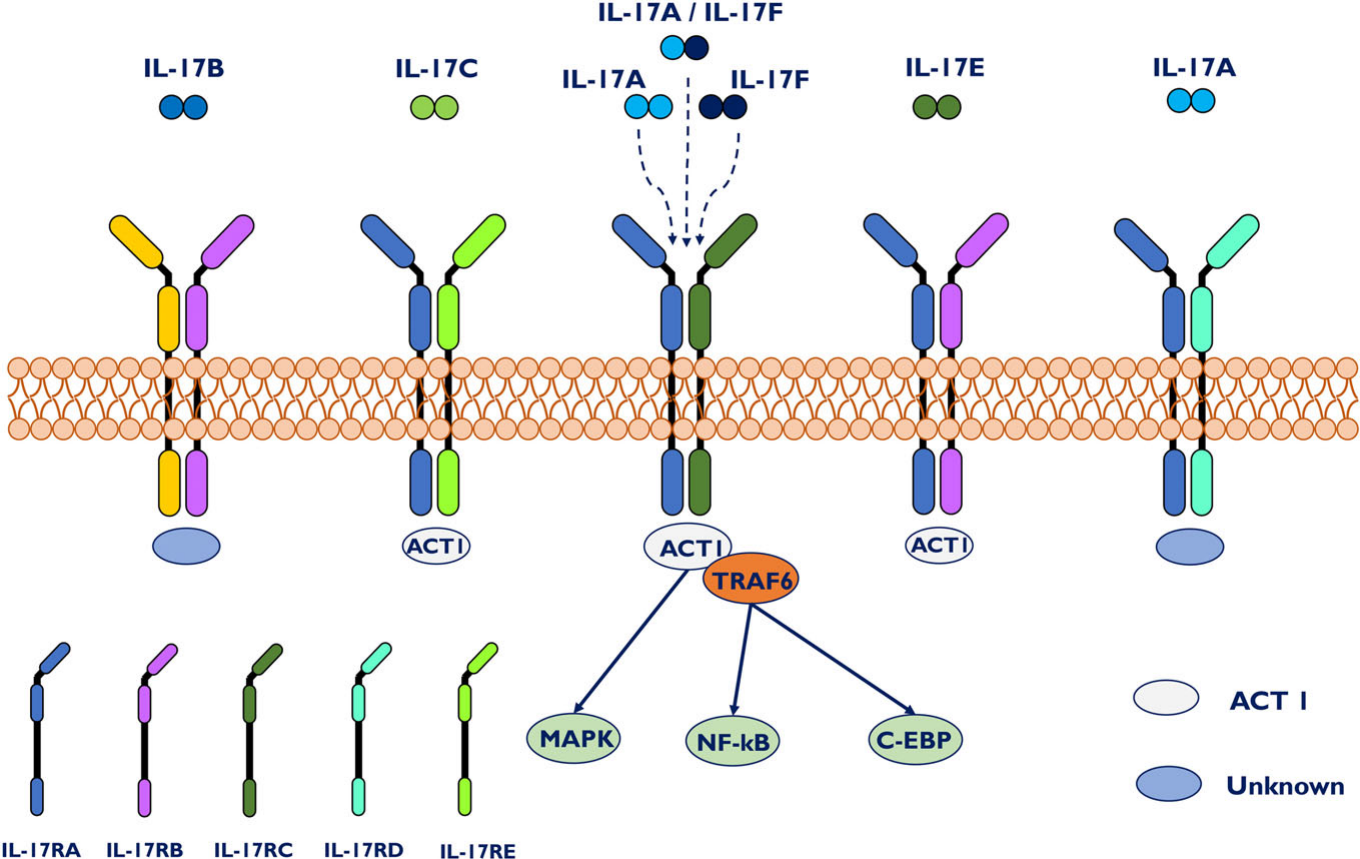
IL-17 family, IL-17 receptors and signal transduction. The IL-17 family is composed of six homologous dimeric proteins (IL-17A to F), while the receptor family consists of five members, IL-17RA to E. IL-17A and IL-17F bind either as individual homodimers or as a heterodimer to a dimeric IL17RA–IL17RC complex. Act1 has been shown to be the key signal-transducing molecule downstream of IL17RA and C, recruiting TRAF6 to further trigger downstream signaling pathways. Act1 has also been identified in IL-17C- and IL-17E-mediated signaling. Specifically, IL-17RA/RD acts only as a receptor for IL-17A/A. IL-17C associates with IL17RA/E, whereas IL-17E binds to IL-17RA/B.

Commonly, the IL-17 family has six members, in alphabetical order, biologically labeled IL-17A through IL-17F (**Figure 1**). Among them, IL-17A is a proinflammatory cytokine participating in the primary responses to fungi and bacterial infections, followed by its close homolog IL-17F (15, 16). Novel research demonstrated that intestinal epithelial cell-derived IL-17D, as the least studied member, serves as a critical factor in regulating group 3 innate lymphoid cell (ILC3) functions and intestinal homeostasis by binding the receptor CD93 (17). These results further expand the biological role of the IL-17 family. IL-17 is a characteristic cytokine of T helper 17 (Th17) cells, and it can also be produced by other innate and adaptive immune cells, including CD8^+^ T cells, γδ T cells, innate lymphoid cells and dendritic cells (18–20). To signal, the biological impacts of IL-17 occur through interactions with its receptor, which is broadly distributed in different tissues and cells of the immune system (21). IL-17RA to IL-17RE are five subtypes of the IL-17 receptor family (IL-17R) (22). Due to the wide expression of IL-17RA and IL-17RC multichain receptors in most cell types, IL-17 has crucial impacts at the local and systemic levels. The activation of IL-17RA and/or IL-17RC signaling upregulates many inflammatory genes, including proinflammatory cytokines and neutrophil-specific chemokines.

Many studies suggest that upregulation of IL-17 contributes to various acute injury microenvironments (23–26). In pulmonary inflammation, overexpression of IL-17 or induction of exogenous IL-17 through the airway induces severe tissue injury in the lung. However, knockout models lacking critical elements of IL-17 signaling are protected from such damage (27). In acute kidney injury, IL-17 triggers neutrophil recruitment to decrease Th17 activation, verifying the role of IL-17 in Th17 cell activation in inflamed tissue (28). Numerous studies have shown that IL-17 is present at sites of the inflammatory microenvironment and in synergistic interactions, amplifying the inflammation induced by other cytokines, including IL-1, IL-6, IL-8, and TNF-a (14, 29, 30). Thus, current studies have provided a deep understanding of IL-17 and novel strategies for acute injury conditions.

## The Role of IL-17 in AP

Novel insights into AP pathophysiology have demonstrated the importance of the immune response on the inflammatory progression of AP (31). Previous studies have shown that the innate immune system is critical in mediating the progression of AP (32, 33). In AP, several types of granulocytes, such as neutrophils, mast cells, macrophages, dendritic cells and platelets, all are the basis for the pathogenesis and development of AP (34, 35). In addition to innate immune cells, T cells are also present in the site of inflamed pancreatic tissue. Immune paralysis occurs, as a result of T cell apoptosis, triggering a breakdown of defense mechanisms during systemic inflammation (36). During AP, cellular damage caused by pancreatic self-digestion can induce the aggregation of CD4^+^ T helper cells producing IL-17 and stimulate an inflammatory response, which is characteristic of this disease. Thus, recruitment of various immune cells will result in further damage to acinar cells as well as multiple proinflammatory cytokines releasing (37).

### IL-17 as a Novel Biomarker of AP

Studies have found that IL-17A upregulates the transcription of proinflammatory and neutrophil-mobilizing cytokines or chemokines in acute inflammatory diseases (38–40). To date, some researchers have confirmed the correlation between IL-17 and AP and suggest that IL-17 is a predictive marker of AP and correlates with the severity of organ failure **(****Table 1****)** (41, 42). The serum level of IL-17 correlates with the severity of AP and is a valuable prognostic factor in assessing disease progression in AP patients (*P* < 0.01) (43). Compared with healthy controls, AP patients had a significant increase in IL-17 during the first 24 hours, with a positive predictive value of 85.3% (44). In a previous study, researchers reported that higher serum IL-17 is an independent risk factor for adverse outcomes in severe acute pancreatitis (SAP) and is related to excessive bacterial load. Furthermore, IL-17 could be applied as a prognostic factor for length of stay, organ dysfunction, and mortality in SAP patients with continuous blood purification therapy (45). A combination of different proinflammatory factors can provide more comprehensive information to diagnose and treat AP. The serum levels of IL-17 in the progression of AP were correlated with CRP and IL-23 levels. Consistent with current clinical evidence, animal models also confirmed that AP induces the expression of various proinflammatory cytokines, including IL-17, which reach a high level during the first week of AP (46). Although IL-17A is associated with the initiation of systemic inflammatory response syndrome in AP, IL-17A may not be the cause of sepsis in the second stage (pancreatic infection and necrosis) (47). Considering its high prognostic value and rapid availability, IL-17 is considered a promising reference marker among single indicators and enhances the predictive validity of AP.

**Table 1:** IL-17 as a novel biomarker of AP with current clinical results.

Research	Year	Patients	Severity of AP	Serum IL-17	P value	Therapy
Jia et al	2014	85	MAP+SAP	170 pg/ml	<0.05	
Chen et al	2019	68	PEP	9.04 ± 2.75 pg/ml	<0.05	
Sotirios et al	2014	150	MAP+SAP	PPV (85.3%)	<0.001	
Gao et al	2018	92	SAP	12.86 ± 2.28 ng/L	<0.001	CRRT
Dai et al	2015	36	SAP	186.4 ± 110.7 pg/ml	<0.05	CVVH
Guo et al	2019	42	SAP	36.6 ± 16.0 pg/ml	<0.05	HVHF
Zhang et al	2018	65	MAP+SAP	28 ng/L	<0.01	
Xie et al	2018	35	AP	91.63 ± 3.33 pg/ml	< 0.01	

SAP,Severe acute pancreatitis; MAP, Mild acute pancreatitis; PEP, Post−endoscopic retrograde cholangiopancreatography pancreatitis; HVHF, High-volume hemofiltration; CVVH, Continuous veno-venous hemofiltration; CRRT, Continuous renal replacement therapy; PPV, Positive predictive value.

### IL-17A Induces Pancreatic Acinar Cell Damage

Clinically, inflammation, edema, necrosis of pancreatic tissue and extrapancreatic organs are the main pathobiological courses of AP. Apoptotic death represents the capacity of acinar cells to recombine their genetic program after the initial phase of mild AP, and necrosis is the main process of SAP cell death (48, 49). Indeed, acinar cells synthesize and release cytokines and chemokines that recruit immune cells, such as neutrophils and macrophages (50). Subsequently, an uncontrollable inflammatory event within acinar cells develops into systemic inflammatory response syndrome (SIRS), which eventually contributes to the high incidence rate and poor prognosis.

Pancreatic resident cells, pancreatic acinar cells and pancreatic stellate cells (PSCs) can produce inflammatory mediators (51). In AP, damaged acinar cells and recruited inflammatory monocytes/macrophages release IL-1β and IL-6 to recruit naïve CD4^+^ T cells into pancreatic tissue, which differentiate into Th17 cells to produce IL-17 acting on IL-17 receptor-positive cells to release various inflammatory mediators exacerbating AP. The IL-17A receptor is a heterodimer complex consisting of IL-17RA and IL-17RC and is broadly expressed in acinar cells, stellate cells, monocytes/macrophages, neutrophils and other immune cells. Mechanistically, IL-17A interacts with acinar cells, infiltrating immune cells, and other cytokines to aggravate the progression of AP and related complications. Acinar cells express DC-SIGN, a phenotype of dendritic cell, promoting the differentiation of naïve CD4^+^ T cells into CD4^+^/IFN-γ^+^ Th1 and CD4^+^/IL-17A^+^ Th17 cells in pancreatic tissues during AP (52).

In addition, neutrophils and macrophages directly induce intra-acinar cellular protease activation and acinar necrosis to determine the severity of AP (53). The inflammatory and immune activation of AP is thought to be characterized by neutrophil infiltration and the production of various proinflammatory cytokines (54). IL-17A strengthens neutrophil recruitment to the area of sepsis, thus ameliorating bacterial clearance (55). Neutrophil consumption assays characterized neutrophils as crucial promoters of acinar cell necrosis and inhibitors of acinar cell apoptosis during AP (56). Thus, IL-17A may amplify the inflammatory cascade during AP by recruiting neutrophils and macrophages to the damaged area, contributing to the severity of AP. Neutrophil extracellular traps (NETs), the second bactericidal mechanism of neutrophils, promotes pancreatic tissue injury and plays key role in the pathophysiological mechanisms of AP (57). IL-17 could enhance reactive oxygen species concentrations and NETs production in neutrophils during AP (58). Leppkes et al. also found that IL-17 recruits neutrophils in a PADI4-dependent manner to induce AP (59). Inhibition of PADIs prevents NETs formation (60). Both animal and clinical studies have shown that IL-17A is elevated during the primary phase of AP and induces pancreatic injury with acinar cell necrosis (61). Notably, IL-17A analogs also damage acinar cells directly and stimulate these cells to secrete inflammatory cytokines and chemokines, thereby amplifying the cascade of AP. The accumulation of inflammatory mediators is responsible for pancreatic acinar cell necrosis and multiple organ dysfunction (62).

### IL-17 Acts in Synergy With Other Proinflammatory Mediators

IL-17A has the ability to act in synergy with other potent proinflammatory mediators to promote the inflammatory response (63). The impacts of IL-17 originate from its capacity to enroll immune cells by producing chemokines and to induce the expression of receptors, particularly the TNF receptor (64), and its synergistic effects with other cytokines. Different proinflammatory factors have their own characteristics in AP. The combination of different proinflammatory factors can provide more comprehensive information for the diagnosis and treatment of AP. IL-17 mainly interacts with nonleukocytic cells, including epithelial cells, endothelial cells and macrophages (65). IL-17 activates the production of other proinflammatory cytokines, such as IL-1, TNF-α, IL-6 and granulocyte-macrophage colony stimulating factor, collectively resulting in an influx of neutrophils (18).

In mice with acute pancreatitis, dendritic cells (DCs) are essential for pancreatic viability and might protect organs against cell stress (66). A recent study reported that Th17 cells from patients with inflammation potently induce the differentiation and activation of DCs that preferentially promote the IL-17 response in a positive feedback loop (67). As an initiator, IL-17 is also involved in T cell-mediated inflammation. In the primary phase of infection or inflammation, IL-17 accelerates proinflammatory cytokine and chemokine release, amplifying inflammatory reactions (68). IL-17 synergizes with other mediators to activate tissue-infiltrating neutrophils and helps eliminate invading pathogenic bacteria. NF-kB, a central molecule, links initial acinar injury to systemic inflammation and perpetuates inflammation in AP (69). IL-17 directly stimulates inflammatory responses or indirectly induces the production of IL-6 by stabilizing IL-6 mRNA through activation of the NF-κB and ERK1/2 MAP kinase pathways (70–72). As a target of IL-17A, IL-6 is necessary for the RORγt-dependent differentiation of Th17 cells (73), suggesting that IL-17A induces positive feedback. In addition, IL-17A triggers the production and release of various cytokines during AP (74), and these cytokines in turn enhance the synergistic secretion of IL-6 and IL-17A in fibroblasts. Yang demonstrated that IL-17 is increased in the early phase of SAP, confirming that the adaptor protein Card9 coordinates IL-17 to balance immune reactions in SAP pathogenesis (75). By activating the IL-23/IL-17/neutrophil axis, IL-17A exacerbates virus-induced AP (76). Thus, the combination of biomarkers will help clinicians develop and adjust the clinical treatment of AP.

### IL-17A Induced Chemokines

Chemokines, a subtype of chemotactic cytokines, are necessary in the recruitment and expression of inflammatory responses (77). Another major target gene of IL-17A is chemokines, particularly C-X-C chemokines, including CXCL1, CXCL2, CXCL4, CXCL5, CXCL8, and others (78). According to a recent study, the mRNA levels of CXCL1, CXCL2, and CXCL5 in acinar cells and CXCL1 in PSCs were increased after stimulation with rIL-17A *in vitro*. Platelet secretion of CXCL4 is a powerful stimulator of pancreatic neutrophil infiltration and tissue damage, and is mainly formed by CXCL2 (79). These chemokines induced by IL-17A are also involved in the proliferation, maturation, and chemotaxis of neutrophils. These results suggest that IL-17 participates in pancreatic injury by regulating the expression of inflammatory cytokines and chemokines during AP (80).

## Interaction Between Gut Microbiota and Immune Cells in AP

**Figure 2:**
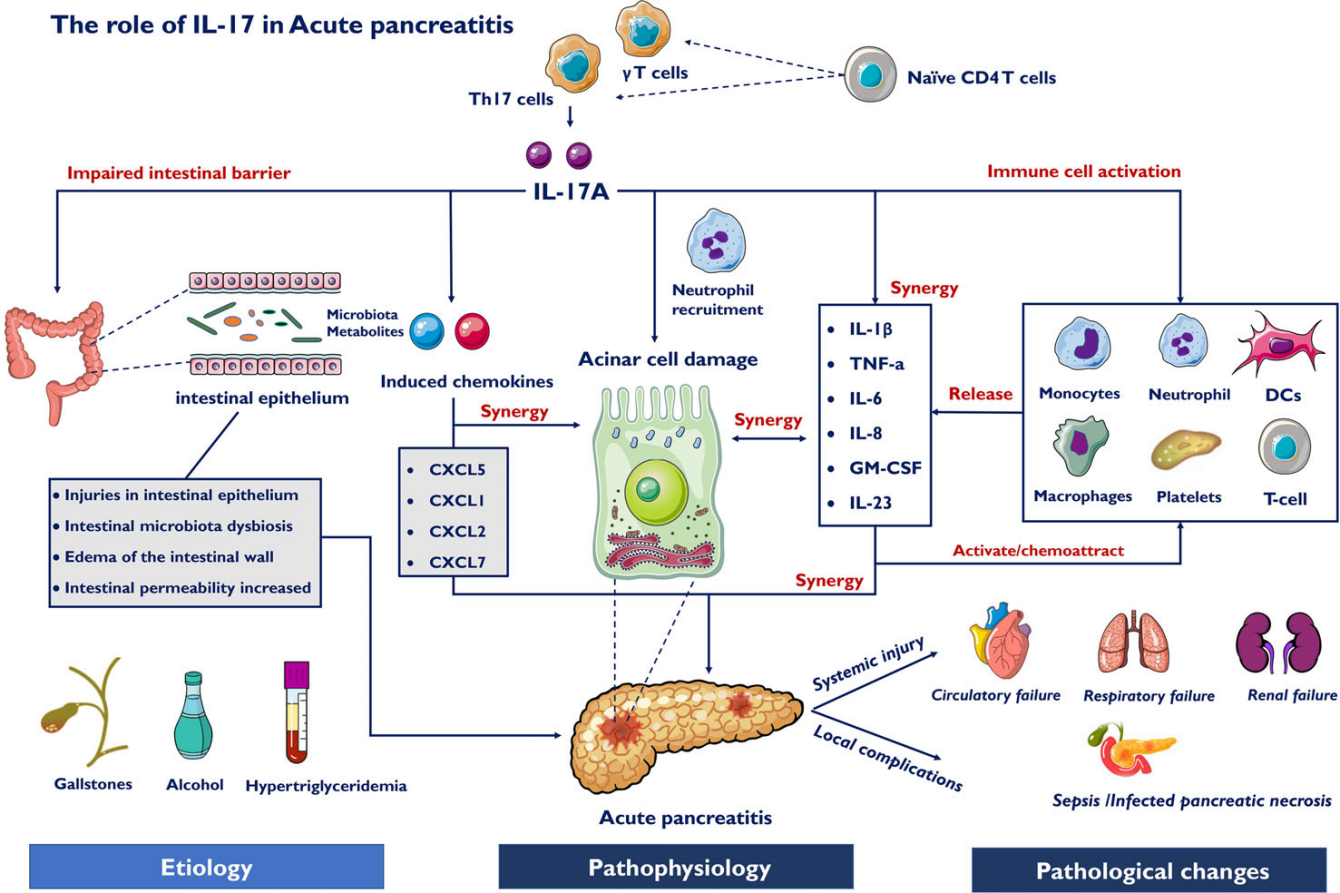
Roles of IL-17 in acute pancreatitis. Etiology for AP include gallstones, alcohol and hypertriglyceridemia, which cause pathophysiological changes of AP. IL-17A induce pancreatic acinar cell damage through synthesizing and releasing cytokines and chemokines which recruit immune cells, such as neutrophils and macrophages. IL-17 also directly induce chemokines and activate innate immune cells, releasing pro-inflammatory mediators. Different inflammatory factors act in synergy with each other to aggravate AP. Furthermore, the intestinal microbiota-derived signals to IL-17 include microbiota alteration, bacteria-derived metabolites and impaired intestinal barrier function. Systemic injury develops early due to IL-17 involving inflammatory response and may precede necrosis, and late secondary organ failure due to infected pancreatic necrosis (IPN) induce sepsis.

Innate myeloid cells are crucial in the activation and differentiation of innate and adaptive immune cells in the intestinal mucosa (81, 82). The inflammatory reaction driven by Th17 cells protects the host from harmful microorganisms, while their overactivation is related to the pathogenesis of intestinal inflammation. A recent study reported that ketogenic diet-related microbiota reduce the levels of intestinal proinflammatory IL-17 (83). Fecal microbiota transplantation (FMT) reduced Th17 cells in mice colonized by donors with Crohn’s disease (84). These studies suggest that the gut microbiome appears to be capable of mediating host immune responses to inflammatory diseases (85, 86). Previous studies demonstrated that dysbiosis of intestinal microbiota was often associated with intestinal barrier dysfunction and metabolic disorders was observed during AP (87). Decreased beneficial bacteria and increased potentially pathogenic bacteria, such as E.coli and Enterococcus, may contribute to dysbiosis of intestinal microbiota and bacterial translocation (88). To confront the microenvironment dysbiosis, the intestine must develop a complex immune protection network including a variety of immune cells. In general, we classified the signals of intestinal microbiota to IL-17 into the following four categories during AP: intestinal barrier function, bacterial components, bacteria-derived metabolites and microbiota-propelled Th17 Cells in AP. Thus, the roles of IL-17 in acute pancreatitis are summarized in **Figure 2**.

### IL-17 Induced Impaired Intestinal Barrier Function

Impairment of intestinal barrier function is an adverse event in AP, which leads to the translocation of gut microbes and endotoxin. The excessive release of inflammatory cytokines during AP is a primary reason for intestinal barrier injury (89). Under AP conditions, the expression of ZO-1, claudin-1 and occludin is decreased, changing the tight connections between cells and promoting the translocation of gut microbes. Damage to the intestinal barrier is related to intestinal microcirculation disturbance, excessive release of inflammatory cytokines, intestinal epithelial cell damage and intestinal microorganism disturbance. As a magnifying agent of the inflammatory reaction, recent research has indicated that increased IL-17 in AP is harmful to maintaining intestinal barrier function and epithelial cells. IL-17 functions at mucosal interfaces, such as the gut epithelial lining, maintaining intact physical barrier and inducing antimicrobial peptides to promote invasion by intestinal bacteria (90). Furthermore, IL-17 results in edema of the intestinal wall, and destruction of the mucosal epithelial barrier *via* expression of CCR6 in enterocytes and mobilization of Th17 cells in the intestines (91). A recent study revealed that IL-17 induces tight junction expression through the ERK-MAPK signaling pathway to alter intestinal permeability (92). Additionally, the level of serum IL-17 is also closely related to bacterial overload, illustrating that the overproduction of IL-17 damages intestinal barrier function, contributing to organ failure in AP.

### IL-17 and Host Gut Microbiota in AP

IL-17A is a key mediator of mucosal monitoring and barrier integrity by promoting the production of antimicrobial factors necessary to contain pathogens (93). IL-17A is involved in inducing an appropriate immune response against resident bacteria in the gastrointestinal tract through a variety of mechanisms, including promoting intestinal IgA responses and the expression of epithelial cell-related innate immune receptors, such as Toll-like receptors (TLRs) and antimicrobial peptides (94). The accumulation of proinflammatory and anti-inflammatory immune cells is regulated by the various commensal microbiota populations within the gut. However, gut dysbiosis can lead to disruptions in immune cell activity. Dysbiosis of the intestinal microbiota contributes to excessive release of inflammatory cytokines, common in both the immune system and intestine in the progression of AP (95). Pathogenic bacteria, such as Escherichia coli and Enterococcus, enriched in AP showed a positive correlation, while beneficial bacteria, such as Bifidobacterium, Lactobacillus and Bacteroides, showed a downward trend (96). Intestinal Th17 cells can be induced by specific microbes, including Escherichia coli and Bifidobacterium. Escherichia coli was positively correlated with the proportion of RORγt+ and IL-17A^+^ Th17 cells in the colon, while the proportion of IL-17A^+^ Th17 cells in the colon of mice lacking only Escherichia coli was significantly reduced (97). Bifidobacterium and Lactobacillus inhibited the expression of IL-6 and IL-17 while facilitating the protein synthesis of major tight junction proteins (98). In addition to strengthening the intestinal epithelial barrier, the microbiome also modulates the immune system and transmits advantages to the host (99). FMT and probiotics, which are both designed to reverse microbial disorders and renew a broader structural status, may provide promising insights into inflammatory and immune response networks, facilitating the recovery of AP.

### IL-17 and Host Gut Microbiota-Derived Metabolites in AP

Metabolites from the microbiota are involved in maintaining intestinal homeostasis or the pathogenesis of inflammation by regulating IL-17 during AP. Short-chain fatty acid (SCFA) attenuated AP, as evidenced by reduced serum amylase and lipase levels and improved pancreatic morphology (100). The regulation of T cells by SCFAs depends on the cytokine environment and immunological microenvironment. In fact, acetate boosts IL-10-producing T cells in a stable state and affects Th1 and Th17 cells in active immune responses. Butyrate depletion appears to play a central role in disease progression towards necrotizing pancreatitis (101). The decrease in butyrate production was associated with the phylum Proteobacteria and the genera Escherichia/Shigella and Streptococcus increasing in patients with AP. Butyrate prophylaxis could mediate the differentiation of CD4+ T cells towards Treg cells, resulting in production of the anti-inflammatory cytokine IL-10 (102). Bile acid metabolites might also regulate the intestinal immune landscape through the balance of Th17 cells and Treg cells (103, 104). Wan found that supplementation with bile acids could relieve pancreatic and intestinal damage, which may be related to the gut microbiome (105). Moreover, the administration of bile acid to an animal model led to a decrease in Th17 cells and an increase in Treg cells in the small intestine lamina propria (103). Tissue-specific anti-inflammatory mechanisms during AP suggest that modulation of gut microbiota-derived metabolite levels could become a potential strategy to improve AP.

### Microbiota-Propelled T Helper 17 Cells in AP

T helper 17 cells are a subset of T lymphocytes with many functions in immune adaptation and have a double-edge sword in pathological and physiological processes. The microbiota is a potent promoter of proinflammatory Th17 cells expressing the lineage-specific transcription factor RORγt (106). Increasing evidence indicates that gut microbiota dysbiosis often occurs in the “second hit” induced by AP gut-derived infection (87, 107, 108). Upon microbiota antigen stimulation, Th17 cells produce several proinflammatory effector molecules which mediate inflammatory cells infiltration and tissue destruction, including proinflammatory cytokines (IL-17A, IL-17F and IFN-γ), chemokines, and matrix metalloproteases (109, 110). Proinflammatory cytokines further stimulate innate immune cells and epithelial cells to produce IL-1, IL-6, and G-CSF, inducing neutrophil recruitment and impairing intestinal barrier function (111, 112). The imbalance between Th17 cells and Tregs is common and relevant to the severity and prognosis of AP. Recent study confirmed that the proportion of Th17/Treg was significantly higher in SAP patients and more pronounced in the multiple organ failure group (*P*<0.05) (113). The homeostasis of the pancreatic microenvironment and rapid response to invaders are tightly regulated by the continuous adjustment between harmful Th17 cells and immunosuppressive Tregs, which establishes an ecological niche for bacterial intestinal border-dwelling bacteria (114). Thus, inhibiting pathogenic Th17 cells and balancing the differentiation of T-cell toward Treg are critical for host defense (115–117).

**Figure 3:**
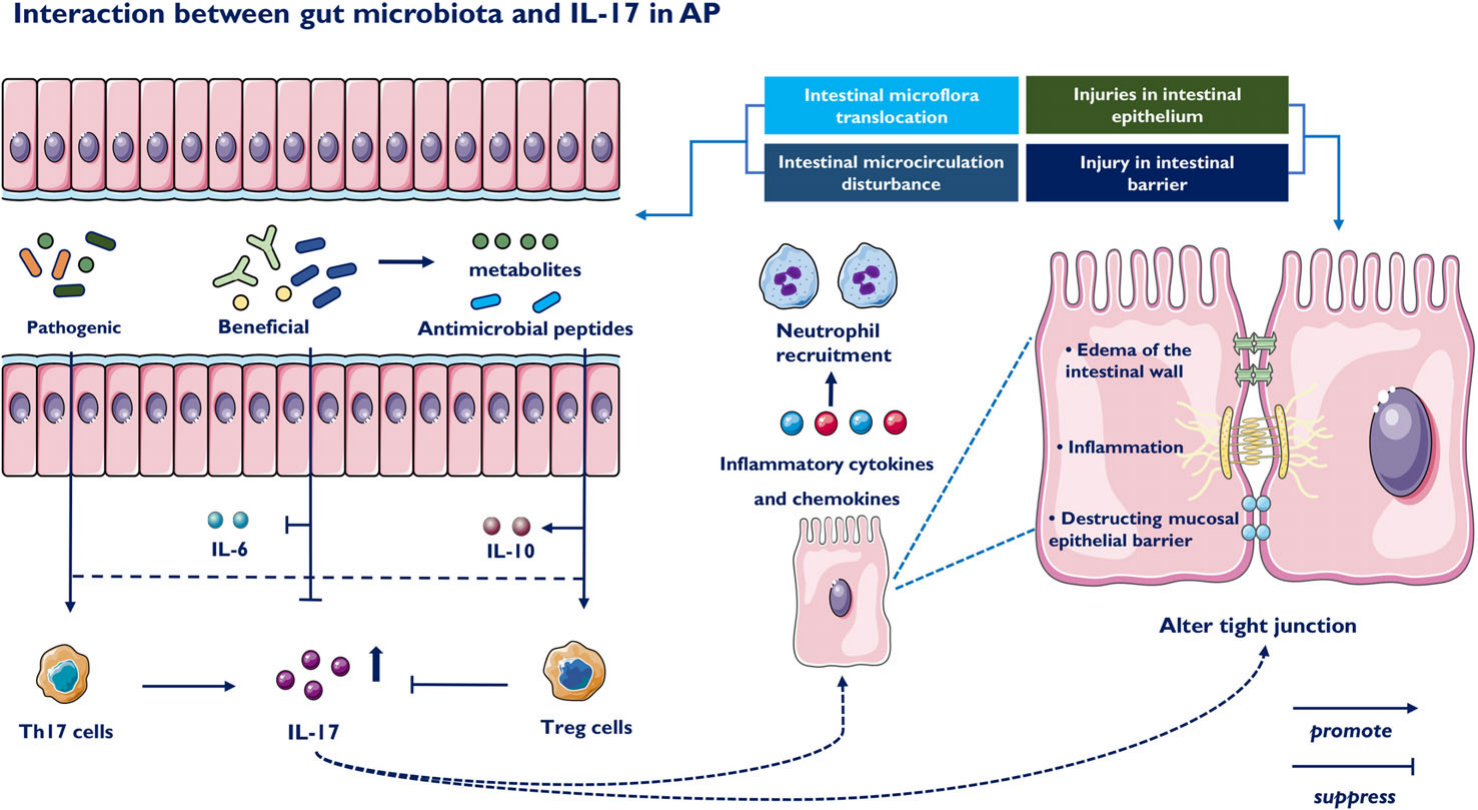
Impact of the gut microbiota on IL-17 and Th17 immune responses. The intestinal microbiota-derived signals to immune cells are classified into the following three parts: bacterial components, bacteria-derived metabolites and intestinal barrier function. Beneficial bacteria suppressed the expression of proinflammatory cytokine IL-6 and IL-17 and promoted the expression of major tight junction proteins. On the contrary, colonization of pathogenic commensal bacteria induces generation of Th17 and downregulates Tregs immune responses. Beneficial bacteria produce short-chain fatty acids, which participate in the generation of Tregs and IL-10 by suppressing proinflammatory cytokines. IL-17 injuries intestinal epithelium, releases inflammatory cytokines and cause intestinal microcirculation disturbance. IL-17 also induces impaired intestinal barrier function, including edema of the intestinal wall and destruction of the mucosal epithelial barrier.

In conclusion, gut microbiota and metabolic disorders in AP often lead to the imbalance of Th17 and Treg, resulting in the increase of IL-17A. Subsequently, IL-17A recruit neutrophils and act in synergy with other proinflammatory mediators to promote the inflammatory response, which destruct intestinal microcirculation and mucosal intestinal barrier. Based on intestinal barrier damage, IL-17A further leads to a pathological bacterial and metabolic translocation, and eventually promote a positive feedback loop to aggravate pancreatic injury (118–120) (**Figure 3**).

## Therapeutic Implications of AP

In AP, a cascade of responses starts with the discharge of endogenous molecules as a result of tissue damage and leukocyte activation. In addition to conservative supportive treatment, drug therapy targeting immunomodulation has achieved promising results. The accumulated discoveries from early clinical research with anti-IL-17 agents yielded strong evidence for the role of IL-17 signaling within the pathophysiology of inflammatory diseases and the potential utilization of these operators in AP therapy (121, 122) Based on the above discussion, this approach represents a strategic therapeutic concept to balance this cascade response.

### Targeting the Th17 Pathway in AP

Moderate blockade of the inflammatory pathway and regulation of the cascade response through different mechanisms may be an effective approach in developing pharmacological drugs for AP. Inhibition of Th17 cell differentiation could reduce IL-17 secretion and delay the progression of AP. Currently, most drugs that block Th17 differentiation are inhibitors targeting IL-23 and RORγt. IL-23 inhibitors are specific antibodies against the p19 subunit of IL-23, including tidrakizumab, guselkumab and AMG139 (123, 124). These antibodies are in clinical trials in patients with autoimmune diseases and have shown remarkable effectiveness for targeting psoriasis. In addition to targeting downstream effectors of Th17 pathways, the primary transcriptional regulator RORγt could be a promising therapeutic target. Several RORγt antagonists are used in ongoing clinical trials, such as GSK805 and TAK-828F, which both have shown prospective efficacy in preclinical models and animal models of inflammatory diseases (125, 126). However, several questions remain about RORγt antagonism, including potential mechanisms of action, key target cells and effects on the homeostatic balance within Th17 cells and Treg cells (127). Furthermore, miR-155 is closely related to the differentiation of CD4^+^ T cells to Th17 cells. Pretreatment of human CD4^+^ T cells with a miR-155 inhibitor might reduce the transition from CD4^+^ T cells to Th17 cells, thereby reducing the secretion of IL-17 and relevant pathological damage to the pancreas (128).

### Targeting IL-17 and Its Receptor in AP

IL-17A released by Th17 and γδ T cells stimulates acinar cells, stellate cells, and various immune cells to augment the release of inflammatory cytokines, recruiting more immune cells, and ultimately causing inflammatory cascades in AP. The suppression of IL-17A secretion or specific blockade of IL-17A receptors may offer a potential therapy for AP. Currently, an anti-IL-17A antibody reduces the impact of IL-17A on pancreatic stellate cells and attenuates pancreatic fibrosis in chronic pancreatitis (129). Several molecules inhibiting IL-17 signaling, including antibodies targeting IL-17A (secukinumab and ixekizumab), have been prepared with the target of IL-17 activity normalization to improve anti-inflammatory reactions. Of note, no obvious safety issue was found with either secukinumab or ixekizumab. IL-17RA inhibitors, such as brodalumab, also block the anti-inflammatory reactions regulated by IL-25 (IL-17E) by inhibiting the proinflammatory impacts mediated by IL-17A, IL-17F and IL-17A/F (130). The combination of IL-17R and different subtypes of IL-17 may upset the anti-inflammatory or proinflammatory balance mediated by IL-17 and produce certain side effects while controlling disease progression. It is possible that anti-IL-17 and IL-17R treatment may be a prospective therapeutic target for AP treatment in the near future.

### Inhibition of IL-17 Synergistic Cytokines

IL-17 and other inflammatory factors, including IL-22, IFNγ, TNF-α, and GM-CSF as well as other synergistic actions, constitute a positive feedback circuit and promote recruitment of inflammatory mediators, aggravating the inflammatory response. TNF-α has been defined as a crucial cytokine that mediates the systemic inflammatory response. Pentoxifyllin, an oral TNF-α antagonist, reduced ICU admissions and shortened hospital stays (131). IL-17 and TNF-α cooperate to induce the expression of receptor activator of membrane protein nuclear factor-κ-ligand, which is involved in tissue remodeling and dendritic cell maturation (132). The combination of IL-17 and TNF-α inhibitors has shown better efficacy in inflammatory diseases. In SAP, ellipticine attenuated NF-κB and MAPK activation in response to IL-17A and TNF-α treatment, inhibited Act1- and TRAF6-mediated NF-κB activation, and blocked the interaction of Act1 with TRAF6 (133). The IL-6/STAT3 signaling pathway is a vital regulator of Th17 and Treg cells by promoting Th17 cells and inhibiting the differentiation of Tregs. Recent studies have shown that three novel small molecule IL-6 inhibitors, madindoline-5 (MDL-5), MDL-16 and MDL-101, significantly suppress IL-17 production (134). Targeting the IL-23–IL-17 axis has shown encouraging results for psoriasis and Crohn’s disease. Pancreas-specific deletion of IκBα leads to nuclear translocation of RelA and diminishes AP induction and trypsin activity (135, 136). (IκB)-ζ, an inhibitor of NF-κB, promotes IL-17 downstream gene expression by inhibiting miR-23b, which acts as a central regulator of the IL-17 signaling pathway. RelA was constitutively activated in mice with pancreatic-specific IκB deficiency, and the gene expression profiling was consistent with NF-κB activation. Targeted knockout of the (IκB)-ζ target gene may effectively inhibit the IL-17-mediated inflammatory response. Thus, the combination of other cytokines will help inhibit the progression of AP.

### Interdicting the AP Cascade Reaction Through Gut Microbiota

As IL-17 exacerbates AP through different mechanisms, pharmacological blockade of the gut microbiota may exert a partial effect on treating AP. Recent studies found that oxymatrine plays an anti-inflammatory role in AP intestinal injury by suppressing Th1/Th17 secretion of cytokines (137). Probiotics have protective functions against intestinal mucosal damage diseases. Oral administration of Enterococcus durans, as a probiotic, to an animal model of colitis confirmed that the GM-related bacterium suppresses the expression of IL-17A and alleviate disease development (138). The Th1/Th2 balance and Treg activity are key elements in the immunomodulatory impacts of probiotics. Recent research has confirmed that certain commensal bacterial species (139), such as segmented filamentous bacteria (SFB), initiate the accumulation of Th17 cells within the digestive tract in numerous species. Furthermore, probiotics changed the intestinal microbial community towards certain advantageous microbes, such as Streptococcus, Lactobacilli and Bifidobacteria, which generated anti-inflammatory metabolites, suppressed Th17 polarization and promoted anti-inflammatory Treg/Tr1 cell differentiation (140). Probiotic strains can potentially inhibit Th17/IL-17 activity by enhancing Treg and/or Th1 subsets (139). However, probiotics have been shown to prevent intestinal infection while unintentionally increasing mortality, leading to early termination of this trial. Fecal microbiota transplantation means transplanting functional bacteria in the feces of healthy controls to the gastrointestinal tracts of patients, and the new microbiota appropriate for the receptors can be modified (141). A few trials have confirmed the ability to improve intestinal function in AP utilizing this strategy (142, 143), but advanced RCTs are still needed.

### The Efficacy of Continuous Renal Replacement of SAP With IL-17

**Figure 4:**
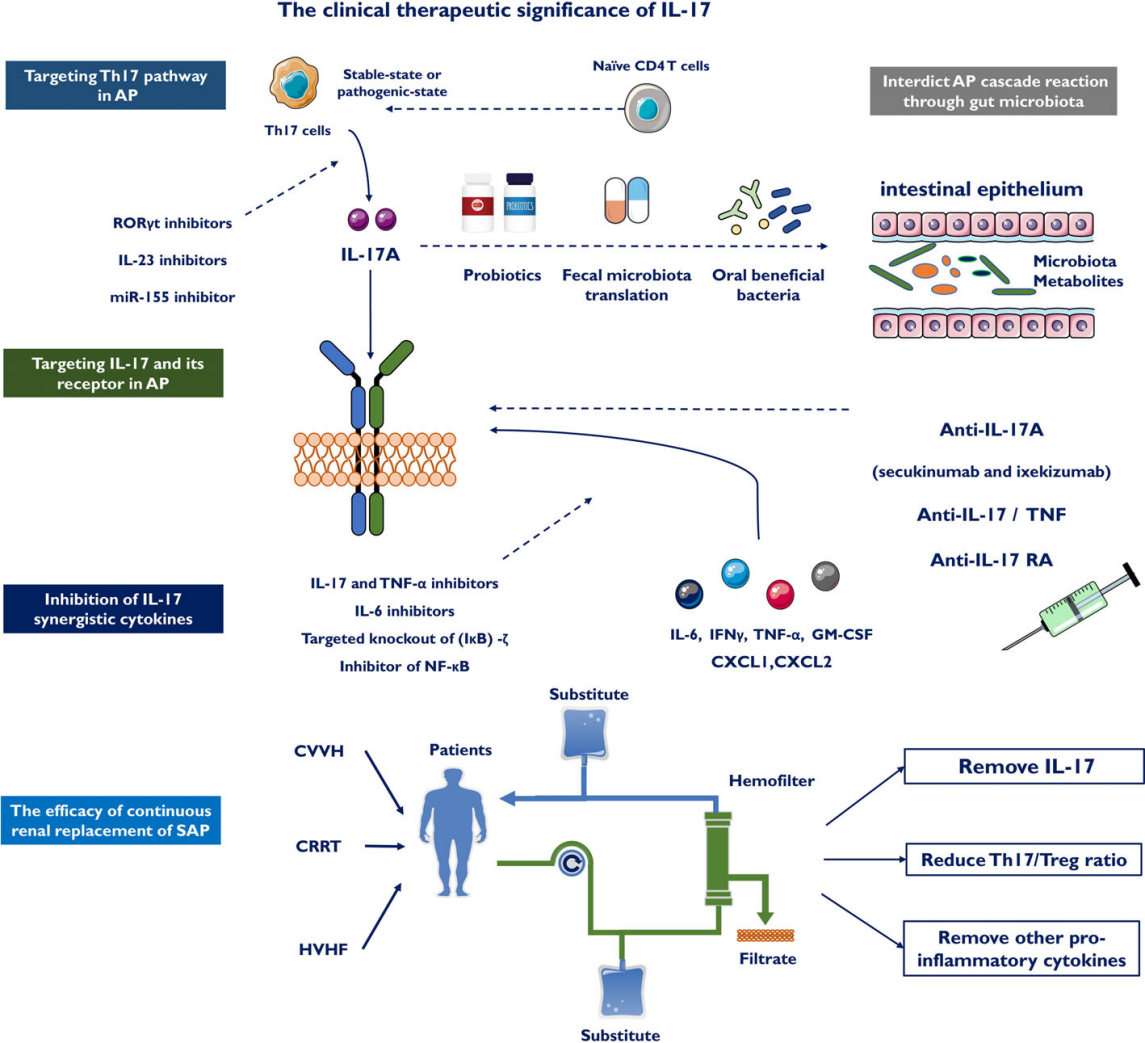
The clinical therapeutic significance of IL-17. IL-17 could boost deeper comprehend of AP and represents a novel immunotherapy for AP by targeting the Th 17 cell/IL-17 immune axis, including targeting Th17 pathway in AP, targeting IL-17 and its receptor in AP, inhibition of IL-17 synergistic cytokines and interdicting AP cascade reaction through gut microbiota. Continuous renal replacement therapy (CRRT), continuous veno-venous hemofiltration (CVVH) and high-volume hemofiltration (HVHF) effectively attenuates the Th17/Treg imbalance and clear serum IL-17 in SAP patients.

Continuous renal replacement therapy (CRRT) is a new model of renal replacement therapy and was first used in the treatment of renal failure. In the comparison of conventional therapy and CRRT, Ning et al. found that IL-17 showed a continuous decrease at 6, 12, and 24 hours after CRRT and was significantly lower than conventional treatment at different time points (*P*<0.001) (144). Dai reported that earlier and higher levels of IL-17 evaluated the extended length of hospital stay, organ failure and death, possibly due to a disruption of intestinal barrier function (45). Researchers found that continuous veno-venous hemofiltration (CVVH) removed IL-17 and other proinflammatory cytokines from serum, improved intestinal barrier function, and relieved systemic reactions. In addition, high-volume hemofiltration (HVHF) effectively attenuated the Th17/Treg imbalance during SAP (113). The Th17/Treg ratio and IL-17 were significantly reduced (*P*<0.05) after HVHF, while no significant change was found in the non-HVHF group. These alterations illustrate that IL-17 plays a key role in the occurrence and development of removing excess inflammatory mediators in SAP patients **(****Figure 4****)**. Therefore, these results suggest the clinical significance of IL-17 and may improve the outcomes of SAP.

## Conclusions

Since the discovery of Th17 cells, the cytokine IL-17 has undergone an increase in considerations and revelations. According to the previous literature, IL-17 causes many acute inflammatory diseases, but the precise mechanisms of its contribution are not fully understood. IL-17 is correlated with numerous cell types, acts on a number of cellular targets in tissue and immune cells, and plays vital roles in innate and adaptive immunity. Dysregulated cytokine systems are generally included in AP, and targeted therapy with IL-17 is of great value. Inhibition of IL-17A and its receptor or simultaneous inhibition of IL-17A and IL-17F contributes to interruption of signaling pathways important for AP development and maintenance. Accordingly, biologics targeting IL-17 contribute to quick and dramatic prompts of systemic symptoms during AP. We believe that IL-17 could lead to a deeper understanding of AP and represents a novel immunotherapy for AP by targeting the Th17 cell/IL-17 immune axis.

## Author Contributions

LeL, GL, and BS conceived of the study and participated in its design. LiL and TL acquired and analyzed study data. TZ, YX, PX, YZ, HC, HT, and XG participated substantially in the organization and coordination of study. GL and HC wrote the manuscript and prepared the figures. All authors contributed to the article and approved the submitted version.

## Funding

This study was supported by The National Natural Science Foundation of China (81871974, 82070658, 81800572) and The Natural Science Foundation of Heilongjiang Province (LH2021H048).

## Conflict of Interest

The authors declare that the research was conducted in the absence of any commercial or financial relationships that could be construed as a potential conflict of interest.

## Publisher’s Note

All claims expressed in this article are solely those of the authors and do not necessarily represent those of their affiliated organizations, or those of the publisher, the editors and the reviewers. Any product that may be evaluated in this article, or claim that may be made by its manufacturer, is not guaranteed or endorsed by the publisher.
